# Targeting the Serine Pathway: A Promising Approach against Tuberculosis? †

**DOI:** 10.3390/ph12020066

**Published:** 2019-04-30

**Authors:** Marie Haufroid, Johan Wouters

**Affiliations:** Laboratoire de Chimie Biologique Structurale (CBS), Namur Medicine and Drug Innovation Center (Namedic), Namur Research Institute for Life Sciences (NARILIS), University of Namur (UNamur), B-5000 Namur, Belgium

**Keywords:** SerB2, phosphoserine phosphatase, HAD, tuberculosis, SerA1, SerC, phosphoserine aminotransferase, phosphoglycerate dehydrogenase

## Abstract

Tuberculosis is still the leading cause of death by a single infectious agent. Effective chemotherapy has been used and improved since the 1950s, but strains resistant to this therapy and most antibacterial drugs on the market are emerging. Only 10 new drugs are in clinical trials, and two of them have already demonstrated resistance. This paper gives an overview of current treatment options against tuberculosis and points out a promising approach of discovering new effective drugs. The serine production pathway is composed of three enzymes (SerA1, SerC and SerB2), which are considered essential for bacterial growth, and all of them are considered as a therapeutic drug target. Their crystal structure are described and essential regulatory domains pointed out. Sequence alignment with similar enzymes in other host would help to identify key residues to target in order to achieve selective inhibition. Currently, only inhibitors of SerB2 are described in the literature. However, inhibitors of human enzymes are discussed, and could be used as a good starting point for a drug discovery program. The aim of this paper is to give some guidance for the design of new hits for every enzyme in this pathway.

## 1. Introduction

Since the introduction of penicillin, a great variety of antibiotics invaded the market between 1940 and 1962 [1,2]. At the same time, most pathogens found a way to select for the resistance to most or all major antibiotics classes such as penicillins, carbapenems, monobactams, cephalosporins, quinolones, aminoglycosides, tetracyclines, and polymyxins [3,4]. Among those resistant pathogens, one growing concern is the apparition of multi-drug resistant (MDR) and extensively drug-resistant (XDR) strains of *Mycobacterium tuberculosis* (Mtb) [3,5,6,7]. Sixty years after the introduction of effective chemotherapy for tuberculosis, the number of cases is higher worldwide than ever before. The threatening part is that there is an increasing number of infections cases with bacteria resistant to major anti-tuberculosis agents [8].

This review overviews tuberculosis (TB) chemotherapy and illustrates the low number of new drugs in clinical trials. In response to the lack of new types of inhibition, the serine biosynthesis pathway is proposed as a possible drug target for the design of new inhibitors. This pathway is essential for bacteria and mammalian cells growth since it is connected to many other metabolic pathways. Serine pathway is composed of three enzymes (SerA1, SerC and SerB2), all considered as possible candidates for drug targeting. The two first enzymes (SerA1 and SerC) are already described but inhibitors have never been proposed. They also seem to be only involved in serine biosynthesis. On the contrary, Mtb phosphoserine phosphatase (SerB2), the third enzyme of the serine pathway, is involved in a virulence mechanism of the bacteria, and a few inhibitors have been reported [9].

## 2. Tuberculosis: Overview

Mtb, also called “the white plague”, was discovered in 1882 as the causative agent of tuberculosis by Robert Koch [7,10]. This bacilli is still the leading cause of death by a single treatable infectious disease, since it kills over 1.5 million people every year and 1.7 million in 2016 alone [11,12,13,14]. Mtb is a member of the *Mycobacterium* family that has over 170 different species. Fortunately, only a few of them can affect human beings. The prevalence of TB in human population is quite high (over a third of global population is infected) but the virulence is lower (less than 10% of patients are actually showing symptoms) [15,16].

**Figure 1:**
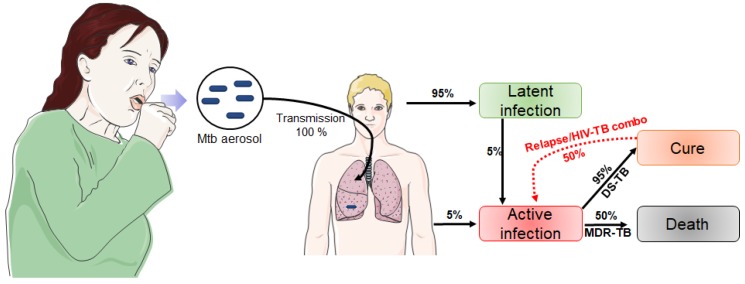
Stages of Mtb transmission and infection cycle showing that 95% of infected patients will have the latent form, and 50% of relapse cases will go directly from latent to active form (only 5% for new cases) [10]. This figure was created using Servier Medical Art templates, which are licensed under a Creative Commons Attribution 3.0 Unported License; https://smart.servier.com.

Virulence and prevalence can be explained by the infection cycle of this bacteria (Figure 1). Once Mtb is in the air, there is a 100% chance of transmission. After transmission, the infection initiates in the lower lung quite efficiently [17]. Most infected people will not show any symptoms (95%) because the bacteria will stay in its latent form. Around 5% of infected patients will directly express the active form of the disease. Fifty percent of those patients may infect other people, while patients with the dormant form are not as contagious. Five percent of dormant patients can go from latent to active infection within years after transmission. This is often due to an immune suppression because of age, concurrent disease, or HIV. Depending on the bacterial strain, there is a 95% possibility of cure when it is treated. However, MDR and XDR strains are harder to cure and show high mortality results (∼50% for MDR-TB and ∼70% for XDR-TB, adapted from [10,16]).

**Figure 2:**
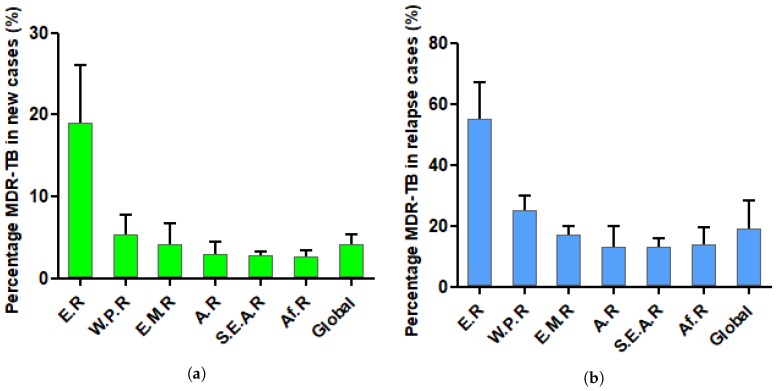
Percentage of MDR-TB in new tuberculosis cases (**a**) and in relapse cases (**b**) [20]. Few data are collected for Africa. E.R., European Region; W.P.R., Western Pacific Region; E.M.R., Eastern Mediterranean Region; A.R., Americas Region; S.E.A.R, Southeast Asia Region; Af.R., Africa Region.

According to the World Health Organization (WHO) report from 2017, there are 4.1 ± 1.3% of Rifampin Resistant (RR) and MDR strains in new tuberculosis cases (Figure 2a). Around 19 ± 8% of previously treated patients show RR/MDR-TB strains when they relapse (Figure 2b). Since most TB cases are located in developing countries, most patients are not reported, and there is a lack of information about the attention they receive, but WHO estimates that half of the patients with MDR-TB and a quarter of those with XDR-TB had or will have successful treatment outcomes [18,19].

Drug susceptible tuberculosis (DS-TB) is currently treated thanks to a combination of four antibiotics: ethambutol, isoniazid, pyrazinamide and rifampin [21]. Those drugs are mostly ineffective against latent TB but very efficient against the active form. To kill every latent bacteria, the treatment should continue over 6–8 months. The duration and cost of this chemotherapy lead to poor compliance from the patient and place a selective pressure on microorganisms [15,22]. Even though the spontaneous mutation ratio leading to resistance is low for Mtb, after 50 years of bad habits in drug administration, globalization, and the spread of HIV, MDR-TB strains became widespread (Figure 2). Indeed, immunodepression due to HIV helps Mtb to infect and grow in lungs. In many less developed countries, HIV and tuberculosis show a mortal synergy.

When first-line drugs are inefficient, second-line drugs such as kanamycin, amikacin, capreomycin, and fluoroquinolones should be used. The majority of those compounds are expensive ($2000–5000 per person against $40 for first-line therapy). They are injectable agents showing high toxicity (nephrotoxicity, ototoxicity, and hepatotoxicity) when administrated together [10]. Moreover, the therapy with second-line antibiotics is four times longer than the one for DS-TB. The chances for a patient in high burden country to comply with this chemotherapy are even lower than for DS-TB [16,23].

Within the context of the Millennium Development Goals, the United Nations decided to start an End TB Strategy to stop and reverse the incidence of tuberculosis worldwide. In 2016, they decided on a new set of goals known as the Sustainable Development Goals with purposes such as “ensure healthy lives and promote well-being for all at all ages”. To reach this objective, TB, AIDS, and other dramatical diseases must be eradicated by 2030. Three pillars are described:

1. integrated, patient-centred care and prevention
2. bold policies and supportive systems
3. intensified research and innovation

**Figure 3:**
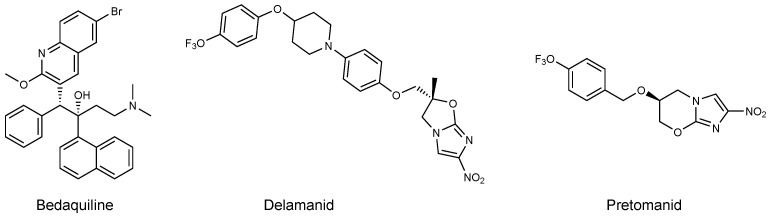
Structure of new compounds in the third phase of clinical trials [14,26].

This review only focuses on the third pillar, which is about research and discovery of new drugs and drug targets to inhibit tuberculosis. Around 200 clinical trials involving drugs against TB are ongoing worldwide [24]. Most of them mix known antibiotics in a new manner to improve the effect, but only 10 new drugs have entered the pipeline. In Phase 3 of clinical trials, there are three compounds, including Bedaquiline (Figure 3), a new diarylquinoline derivative from Janssen Pharmaceutica. This drug is an inhibitor of F${}_{1}$F${}_{0}$-ATP synthase of Mtb and blocks the production of ATP needed for growing bacteria [25]. It was approved under Food and Drug Administration’s accelerated-approval regulation in 2012 as a last resort drug for the treatment of patients with MDR-TB for whom treatment with known antibiotics regimens is ineffective [26,27]. Bedaquiline has also been approved by the European Medicines Agency in 2014 but trials are still ongoing to determine its long-term effect on patients [20].

Delamanid is an imidazooxazole derivative developed by Otsuka Novel Products Gh and was approved in 2014 by EMA for the treatment of MDR-TB [26]. It has strong side effects similar to Bedaquiline, but clinical trials are almost complete. Similar to most anti-tuberculosis drugs, Delamanid targets bacterial cell wall by the inhibition of mycolic acid biosynthesis [28,29]. Only months after the approval of both Delamanid and Bedaquiline, resistance was already reported in a patient [30,31].

Pretomanid is a nitroimidazole with a new mechanism of action developed by TB Alliance [14]. This compound is active on both replicating and non-replicating bacteria and inhibits mycolic acid biosynthesis. It also induces some respiratory toxicity within bacteria [32]. Safety and efficacy of this compound was assessed, and it is now tested as part of a regimen to treat MDR and XDR strains [18]. Other new compounds have reached Phases 1 and 2 in clinical trials, such as Delpazolid, PBTZ169, SQ109, Sutezolid, GSK-3036656, OPC-167832, and Q203. It will take years to show if they are efficient. The question is: Why have only a few drugs entered the TB clinical pipeline?

First, when a new drug is to be made, pharmaceutical companies test their chemolibrary of compounds on the target (the bacteria). However, results from GSK and others showed that this strategy is disappointing and financially unsustainable [33]. In comparison to compounds developed to treat other diseases, antimicrobial agents have different properties. They do not obey Lipinski’s “rule of five”, rules that Christopher A. Lipinski proposed to define the optimal drug-like features of new compounds [34,35]. However, pharmaceuticals libraries of compounds are mostly small “drug-like” scaffolds used to make drugs with those defined properties. It is then not surprising that the screening of those molecules has failed to procure new leads [35]. Indeed, antibiotics should possess a lower lipophilicity to cross the membrane, a higher molecular weight than usual and a larger total polar surface area [10].

This is why GSK and other research groups decided to take a target-based approach. This strategy already showed success with other pathogens such as *Staphylococcus aureus* or *Streptococcus pneumoniae* and can be helped by the decoding of Mtb genome sequence in 1998 [33,36]. Cole et al. [36] sequenced the genome of Mtb. They discovered that 40% of predicted genes had unknown functions for the metabolism of the bacteria. In an attempt to find a large spectrum compound inhibiting targets present in every bacteria, they saw the complexity in the discovery of a minimal set of genes required for bacterial growth and life [37]. In this context, many metabolic pathways and enzymes catalyzing them were proposed as potential targets for drug discovery. This review only focuses on one, the serine pathway.

## 3. Targeting the Serine Pathway

**Figure 4:**
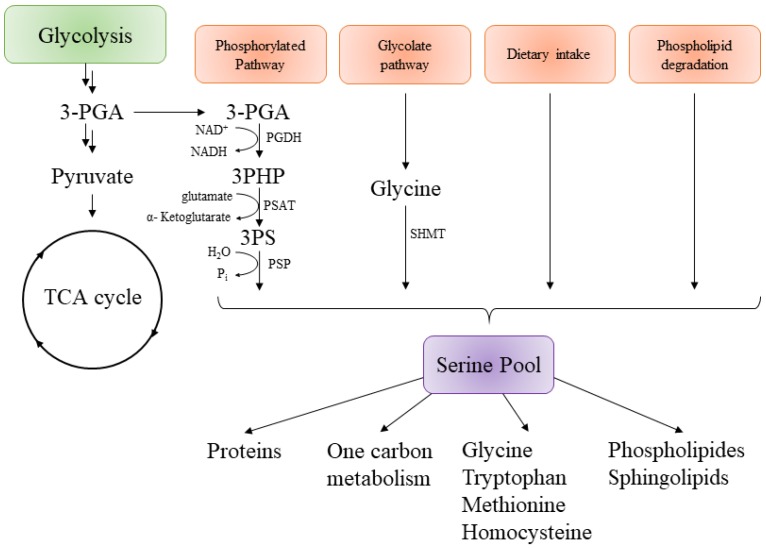
General representation of serine biosynthesis in different organisms and its connection with different metabolic pathways. 3-PGA, 3-phosphoglycerate; 3PHP, 3-phosphohydroxypyruvate; 3-PS, 3-Phospho-l-Serine; PGDH, Phosphoglycerate dehydrogenase; PSAT, Phosphoserine aminotransferase; PSP, Phosphoserine phoshatase; SHMT, Serine hydroxymethyltransferase; Pi, PO${}_{4}^{3-}$; TCA, tricarboxylic acid cycle or Krebs cycle [46].

The serine biosynthesis pathway is required for growth and at the center of other metabolic and biosynthetic pathways (Figure 4). Serine is a non-essential amino acid, meaning it is not essential to provide it by food. Cells can produce it from scratch thanks to a carbon source such as glucose [38]. However, it has also been shown that a supplement in serine within growth media could improve the growth rate in any cell culture [39,40].

In all organisms, L-serine can be derived from four different sources (Figure 4), the first one being by the biosynthesis from the glycolytic intermediate 3-phosphoglycerate (3-PGA) [41]. The second one is from glycine, another non-essential amino acid that is supplemented to insure sufficient quantities of serine for cells. The third one is by dietary intakes via a serine transporter and the last one is by protein and phospholipid degradation. The contribution from each road to the production of the serine pool is not well-described, but results from isotopic markers (or other experiments) suggest that the biosynthesis via the phosphorylated pathway is the major source of serine in mammalian cells and bacteria [42,43,44,45]. L-Serine can then be used to produce pyruvate, glycine, cysteine, D-serine (a neurotransmitter), one-carbon metabolism, proteins, purines, or pyrimidines.

Interestingly, some bacteria such as *Escherischia coli* and *Salmonella typhimurium* can only produce serine via the phosphorylated pathway. One single mutation in proteins involved in biosynthesis directly leads to serine auxotrophy and large growth defects [47]. Other experiments suggest the same thing for mycobacteria such as Mtb. Even though genetic studies are complicated for mycobacteria [48,49,50,51,52,53], Tuffariello et al. showed the possibility for serine auxotrophs Mtb strains to grow in serine supplemented media [54].

**Figure 5:**
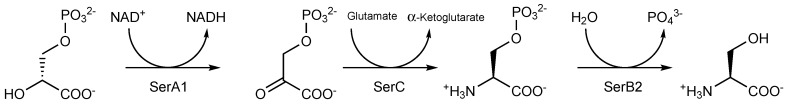
Representation of serine phosphorylated pathway of Mtb in which each step is catalyzed by a different protein. SerA, Phosphoglycerate dehydrogenase; SerC, Phosphoserine aminotransferase; SerB2, Phosphoserine phosphatase [48,57,58].

The serine pathway is composed of three different proteins catalyzing three sequential steps in the synthesis of L-serine (Figure 5). All three were found to be essential for *mycobacterium*’s growth in H37Rv strains by Sassetti et al. [37]. The first enzyme in this pathway is phosphoglycerate dehydrogenase (PGDH or SerA1), and it catalyzes the oxidation of D-3-phosphoglycerate into 3-phosphohydroxypyruvate with NAD${}^{+}$ as a cofactor for the reaction [55]. The second enzyme is a phosphoserine aminotransferase (PSAT or SerC), which converts phosphohydroxypyruvate into L-3-phosphoserine with glutamate being an amine donor [56]. Finally, the phosphoserine phosphatase (PSP or SerB2) dephosphorylates L-3-phosphoserine into L-Serine.

This pathway is well conserved among different organisms and can be found in bacteria, mammals, humans, and plants [46,59,60,61,62]. This pathway is always composed of the three previously cited enzymes. Structural differences can occur from one specie to another, i.e., human phosphoserine phosphatase is only composed of the PSP domain while SerB2 possesses two regulatory domain. Human phosphoglycerate dehydrogenase possesses a mutated ACT domain which avoid product inhibition as opposite to the one of *E. coli* and Mtb [55,63]. Every enzyme from this pathway is a potential drug target. SerA1 and SerB2 are the most described in the literature.

### 3.1. *Mycobacterium tuberculosis* Phosphoglycerate Dehydrogenase SerA1: Old But Gold?

SerA1 gene coding for type I phosphoglycerate dehydrogenase was considered an essential gene in *Mycobacteria* [37]. This enzyme catalyzes the first step of the serine pathway and is part of the 2-hydroxy acid dehydrogenases family. Enzymes from this family are specific for D-configurated substrates [64]. Catalysis of the reaction can occur both ways, with an equilibrium favored in the phosphohydroxypyruvate reduction [63].

**Figure 6:**
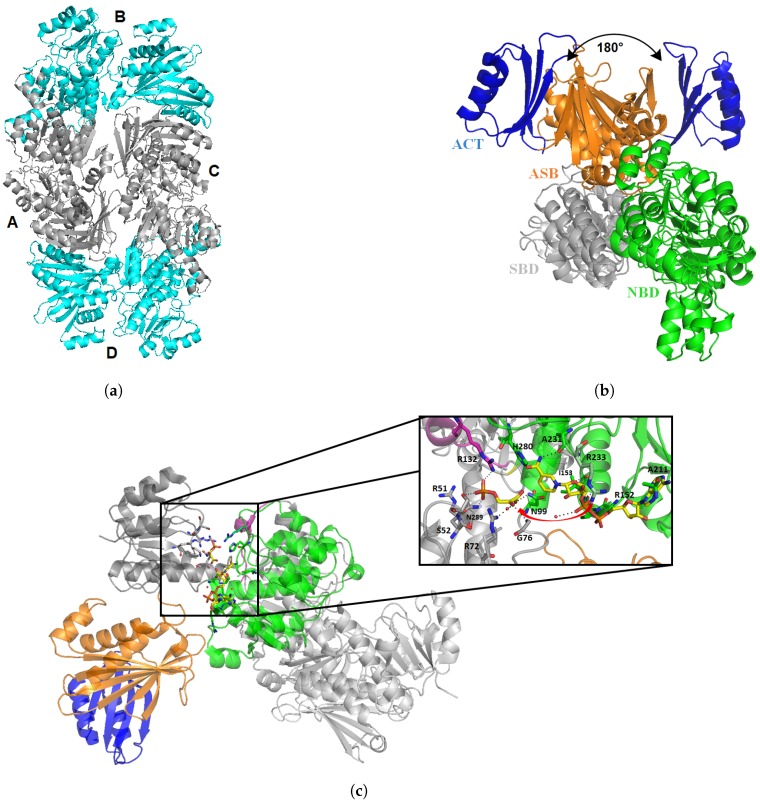
Structure of Mtb PHGDH (SerA1, 1YGY). The tetrameric form of SerA with two monomers in blue (B and D), and two 180° shifted monomers in grey (A and C) (**a**). View of one monomer (**b**) with domain ACT in blue, the Allosteric Substrate binding domain (ASB) in orange, the Substrate Binding domain (SBD) in grey, and the Nucleotide Binding domain (NBD) in green. Superimposition of both monomers shows a 180° rotation of ASB/ACT domain. Structure of SerA1 with the substrate 3-phosphohydroxypyruvate (PDB: 3DDN, (**c**)) and generated NADH (from human PHGDH, 2G76) bound to SBD and NBD, respectively. An helix (in pink) from NBD from the other monomer is directly interacting with the substrate in SBD.

It was first crystallized by Dey et al. in 2005 (PDB: 1YGY), and since then two other structures were deposited by the same team [65,66]. SerA1 presents two molecules within the asymmetric unit and forms a homotetramer by symmetry (Figure 6a). Each monomer is constituted of four major domains: the substrate binding domain (SBD), the nucleotide binding domain (NBD), the allosteric substrate binding domain (ASB), and the regulatory domain, also called ACT (Figure 6b).

NBD is a variation of the Rossman fold with seven β strands and seven α helices, which binds the NAD${}^{+}$ cofactor. This domain is directly connected to the SBD, which contains five parallel β strands and five helices. All together, SBD and NBD form the catalytic cleft. Kinetics and fluorescence resonance energy transfer experiments were performed by Burton et al. [67]. They showed that there is a precise order for substrate and cofactor binding. Indeed, substrate of the reaction (phosphohydroxypuruvate) binds first the SBD, but will not be in the proper orientation for the reaction. Then, NADH binds the NBD, and few amino acids (e.g., R233) will move to a new position for the reaction. This induced fit will force the substrate to be correctly orientated for the reaction by salt-bridges with different arginines residues (Figure 6c).

It was suggested that a movement of ASB could occur in order to let the cofactor in and out of the enzyme. Indeed, superimposition of both monomers shows a 180° rotation of ASB and ACT domains in comparison to the other domains. This shift induces an asymmetry of the tetramer composed of two regular monomers, and two shifted ones (Figure 6a). This rearrangement is not observed in *E. coli* PGDH, and is due to a segment of three glycine residues (316–318). Mutation of G318 leads to a five-fold decrease in protein activity, but mutation of two glycine shows an increase in substrate affinity [66].

**Figure 7:**
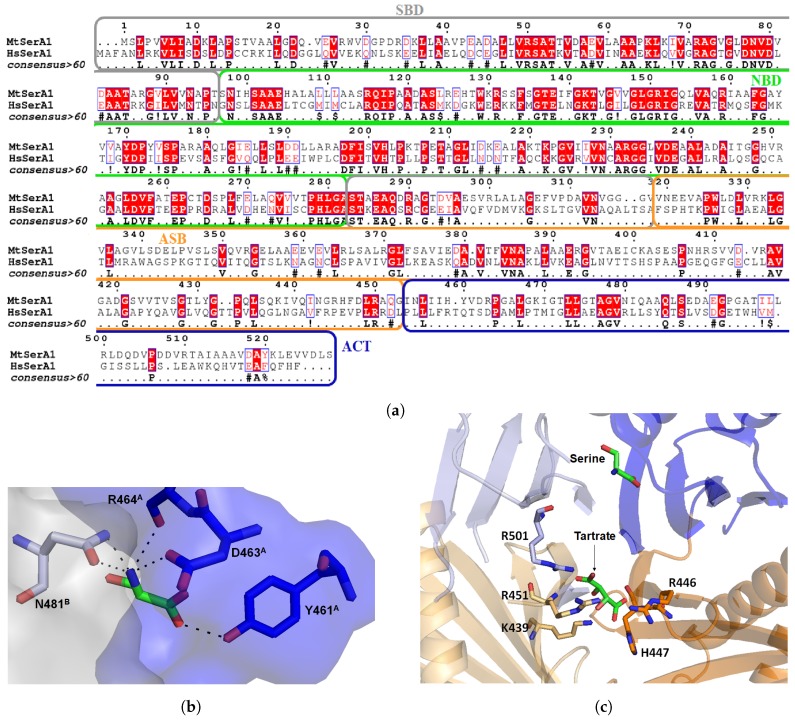
Sequence alignment of MtSerA1 and human PHGDH showing 33.5% overall identity and 50.7% similarity (**a**). Serine binding site within ACT domain of MtSerA1 with the ACT domain surface in blue and the one from another monomer in light grey (**b**). Anion/substrate binding site with a tartrate molecule interacting with different arginines and other positively charged amino acids (**c**).

A promising approach to inhibit SerA1 would be to target regulatory, or substrate binding domains. ACT domain can bind L-serine, the product of the pathway in order to inhibit the reaction in an allosteric manner (Figure 7b). Indeed, structure with L-serine (PDB: 3DC2) shows that it selectively binds Y461, D463, R464 from the ACT domain of monomer A and N481 from the same domain in monomer B. This amino acid binding site is tight, thus only small inhibitors can reach it. Human PHGDH also possesses this domain but like in most mammals enzymes, amino acids were mutated in order to lose this regulatory activity (Figure 7a) [55]. A close look to sequence alignment shows that residues 458–464, which form the serine binding site, are different. In particular, R464 is mutated into an aspartate in the human form, and Y461 into a glutamine. A small selective ligand could potentially be designed to interact with polar residues in order to inhibit specifically the bacterial enzyme.

The other allosteric site, the ASB domain was first described as the intervening domain or anion-binding site and seems to be unique to this enzyme [66]. It contains 150 amino-acid and has a αβααββ motif. In all crystal structures of SerA1, a tartrate is interacting with this domain (Figure 7c). It interacts with positively charged residues (arginines, lysines and histidine) from two different monomers. Burton et al. experimentally showed that the enzyme could be inhibited in presence of high substrate concentration, and the latter can bind this effector site. Human PHGDH also possesses this domain but this allosteric mechanism was never demonstrated. Mutation of three amino acids (K439, R456 and R501) from this domain eliminated the regulatory action of both ASB and ACT domains. Interestingly, on those three mutated residues, two are not conserved in the human form. This may explain the fact that human PHGDH is not inhibited by substrate and serine. Moreover, it means that a ligand able to interact with this highly positively charged domain should be able to selectively inhibit the enzyme [67,68].

#### Inhibitors of Phosphoglycerate Dehydrogenases

**Figure 8:**
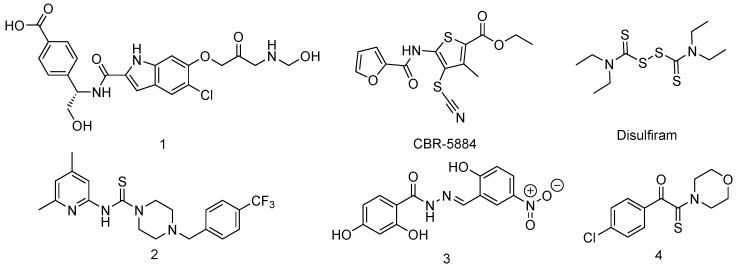
Structure of currently known inhibitors of human PHGDH that could be used in order to design inhibitors of MtSerA1.

There are no known inhibitors of MtSerA1 in literature, but inhibitors of the human PHGDH, which is structurally similar, have been described (Figure 8) [69]. They could be used as a starting point for hit discovery against MtSerA1. For example, compound **1**, an indole derivative, was discovered in 2015 by the group of AstraZeneca [70]. It has been shown that the indole part of the inhibitor interacts with the NBD and that the carboxylate moiety interacts with the SBD. Those domains are mostly well conserved between human and Mtb enzymes (39% identity and 55.6% similarity). Moreover, this inhibitor was co-crystallized with human PHGDH and interacts with Y173, D174, L192, S211, and R235, which are conserved in the Mtb enzyme. This compound has a Ki of 0.18 μM and an IC${}_{50}$ of 1.4 μM for human PHGDH [71]. It was discovered during a fragment-based lead generation. Since MtbSerA1 is able to crystallize, a similar approach could be applied.

Compounds CBR-5884 and disulfiram were first described by Mullarky et al. [72] in 2016 and seem to be non-competitive inhibitors that interact with a cysteine from allosteric sites. The authors showed that these molecules were able to affect the oligomerization state of PHGDH and stabilize it as an inactive dimer. The compounds were discovered during a high throughput screening of 800,000 compounds and could be a good starting point to design selective inhibitors of MtSerA1 that target ASB and ACT domains.

Compound **2** was discovered by the group of Pacold et al. [73] in 2016 after a screening of 400,000 compounds from NIH Molecular Libraries Small Molecule Repository. This compound had a good inhibitory activity (IC${}_{50}$ of 2.5 μM) and was found inactive against other dehydrogenases. Good pharmacokinetics and ADME properties make it a promising candidate. Wang et al. [74] discovered compound **3** in 2017 by structure-based approach. This compound could bind an allosteric site of human PHGDH with IC${}_{50}$ of 34.8 μM and K${}_{D}$ value of 0.56 μM. It shows activity against PHGDH-amplified breast cancer cells in mice. The same year, Ravez et al. discovered compound **4** after a screening of 336 molecules from a fragment library and in-house collection. A pharmacophore could be designed after those experiments and a new non-competitive inhibitor was synthesized with an IC${}_{50}$ value of 30.9 μM on human enzyme. K${}_{i}$ values were determined to be around 40 and 27 μM against two substrates (3-PG and NAD${}^{+}$). Rapid dilution experiments suggest that this inhibitor is covalent [75].

All the described above compounds could be used to facilitate the discovery of new inhibitors of MtSerA. Their activity against desired enzyme could be assessed, and crystallization assays or docking experiments could be done in order to understand their binding mode.

### 3.2. *Mycobacterium tuberculosis* Phosphoserine Aminotransferase SerC

**Figure 9:**
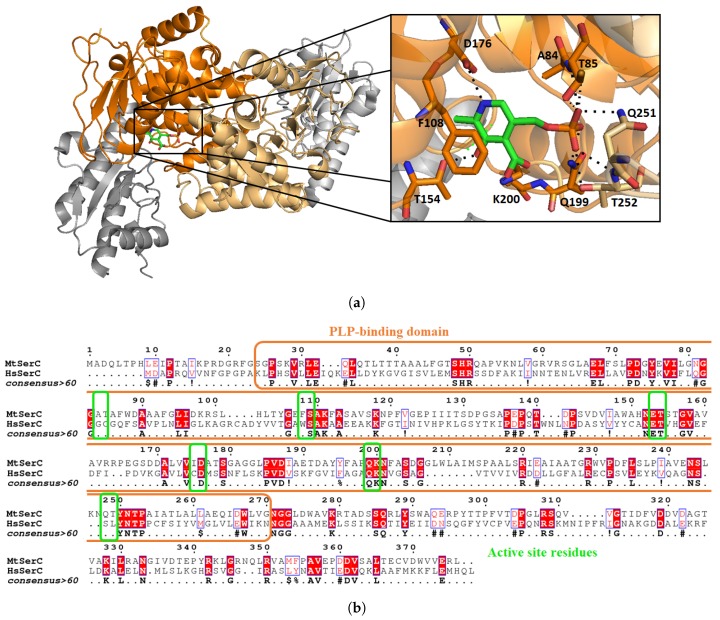
Structure of MtSerC homodimer (2FYF) with the substrate binding domains in orange. Zoom on the active site which contains SerC cofactor pyridoxal 5′-phosphate (PLP, (**a**)). Sequence alignment of MtSerC and human PSAT with substrate binding domain in orange and active site residues in green. The percentage identity between both proteins is 23.6% and the similarity of 40.9% (**b**).

Phosphoserine aminotransferase SerC from *Mycobacterium tuberculosis* is scarcely discussed in the literature. There is only one paper from Coulibaly et al. [56], who determined the crystal structure of SerC with its pyridoxal 5’-phosphate (PLP) cofactor at a resolution of 1.5 Å (Figure 9a). Overall sequence identity of Mtb SerC with the human PSAT is low (23.6%). SerC has the same structural characteristics compared to other known aspartate aminotransferase. A family with a conserved active site, and small differences between residues binding the substrate phosphoryl group (Figure 9b). For example, Q251 and T252 are mutated into serine and leucine in the human form, and A84 and T85 are replaced by glycine and cysteine. Those differences could be used in the design of selective inhibitors which would interact with the polar residues involved in phosphoryl stabilization.

SerC folds as a homodimer and possesses two α/β domains, the first one being a seven-strand parallel β sheet and seven α helices. The second smaller domain is constituted by a three-strand anti-parallel β sheet with three α helices. Within the active site, PLP is not linked covalently to K200 as opposite to other known structures of PSAT enzymes, but its position and orientation in the active site is conserved. To the best of our knowledge, no inhibitors of MtSerC or human PSAT have been reported so far.

### 3.3. *Mycobacterium tuberculosis* Phosphoserine Phosphatase SerB2: A Promising Therapeutic Target?

One of the most eminent mechanism for regulation of molecular processes within cells is the protein phosphorylation and dephosphorylation equilibrium [76]. Biophysical properties of the phosphoryl group are large and diverse, making it a good candidate for protein structure disturbance [77]. It is also well known for prokaryotes to secrete phosphatases within host cells as for example tyrosine phosphatase secreted by Mtb (MPtpA and MPtpB) [78,79].

The fact that a phosphorylation or dephosphorylation reaction can alter the host defence mechanism and inhibit immune response is not surprising [80]. Lately, a few reports showed the importance of secreted phosphatases in the cellular shut down process within macrophages [79,81,82,83,84]. Comparison of *Homo sapiens* and Mtb genome sequences showed differences between genes of similar pathways, such as the lipid and carbohydrate metabolism and amino acid metabolism. In this last case, the phosphoserine phosphatase encoding genes serB2 and serB (Rv3042c, Rv0505c) were declared “important drug targets”, meaning that their inhibition could lead to bacterial death [85]. Indeed, Sassetti et al. [37] constructed a large library of transposon insertion mutants, and obtained mutated bacteria containing a copy of the desired transposon in its genome. After application of strict criteria, they identified different metabolic or biosynthetic pathways required for Mtb’s growth. Among all these pathways, they showed that mutation in the gene coding for SerB2 inhibited bacterial growth. In contrast, SerB1 was found to be non-essential for Mtb viability [58]. This review only focuses on SerB2.

**Figure 10:**
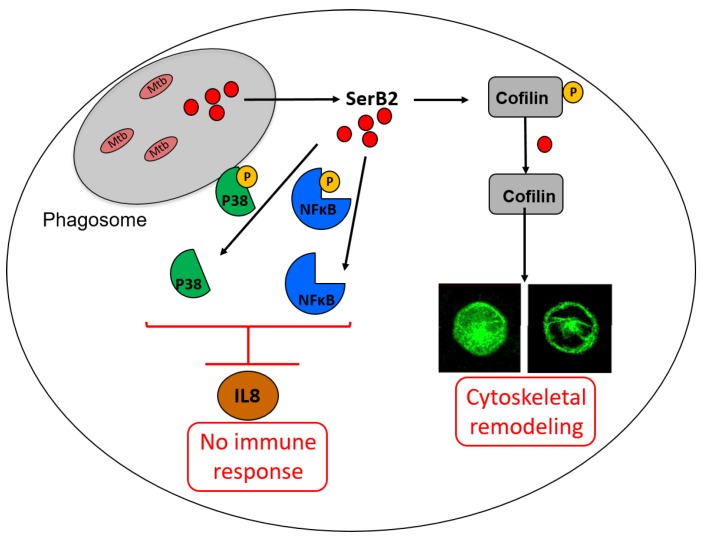
Illustration of how SerB2 can be secreted within macrophage cytosol to disturb immune response and cause cytoskeletal remodeling (adapted from [92]). IL-8, Interleukin-8, an immune mediator; p38, mitogen-activated protein kinase p38, which regulates the expression of many cytokines.

Mtb phosphoserine phosphatase SerB2 was described by Arora et al. [57] and Yadav et al. [58] in 2014. They hypothesized that, in addition to its usual role in the serine pathway, SerB2 interacts with the host signalling pathways similarly to *P. gingivalis* phosphoserine phosphatase (SerB653) [86,87,88,89,90,91]. This was confirmed by Shree et al. [9] in 2016, who performed a complete mechanistic study of SerB2. They demonstrated that MtSerB2 is overexpressed and mostly secreted within the cytosol of infected macrophages (THP-1 cells) by Western blotting, immunodetection and confocal microscopy experiments. Secretion of SerB2 within macrophage induces cytoskeletal remodeling as shown by confocal microscopy (Figure 10). This remodeling leads to a necrotic environment that is highly favourable for Mtb growth. Interaction between SerB2 and cofilin was demonstrated by Western blot, immunoblot and GST based pull down assay. They also showed that the protein interacts with NFκB and P38. Western blot also showed that SerB2 is able to dephosphorylate the above mentioned enzymes in order to inhibit the expression of interleukin 8 (IL-8), an immune mediator. Those protein–protein interactions did not occur with an inactive mutant of MtSerB2 or in the presence of clofazimine, as shown by Western blot and pull down assay. The interaction between this known antibiotic and the enzyme was demonstrated by activity assay and ITC. Finally, the expression of IL-8 was significantly increased once MtSerB2 was inhibited by clofazimine or mutated. MtSerB2 can thus be described as an invasive secreted virulence factor used for immune invasion and evasion [92].

MtSerB2 belongs to the haloacid dehalogenase (HAD) superfamily of enzymes, a family containing more than 19,000 unique sequences of members represented in all types of organisms [93,94]. Most members of this family transfer a phosphoryl group during catalytic activity. Members of this family are ATPases (around 20% of the family) and phosphomonoesterases (around 79% of the family) [95,96,97]. Dephosphorylation occurs thanks to the formation of a phospho-aspartate intermediate. The latter is then substituted in an acid-base catalysis reaction. Most of the enzymes within this superfamily are involved in defence pathways, meaning that they contribute to the detoxification or the degradation of secondary metabolites by-products in order to conserve cell integrity [98].

**Figure 11:**
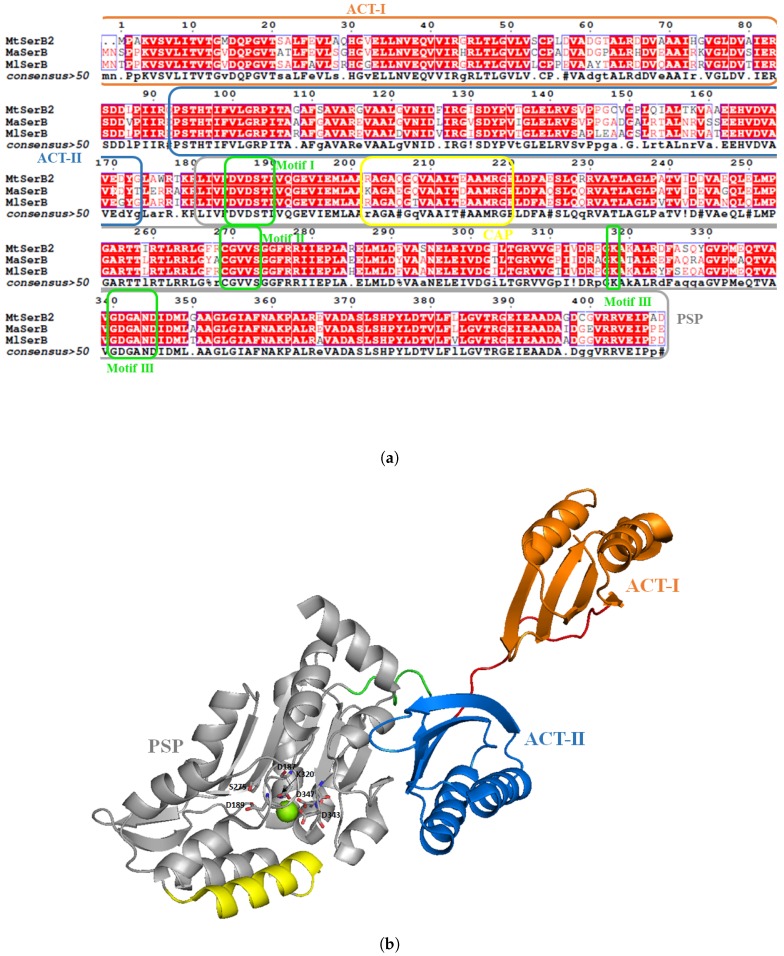
Similarity between phosphoserine phosphatases from *Mycobacterium tuberculosis* (Mt), *avium* (Ma) and *leprae* (Ml) (**a**). Red color in the sequence means that residues are strongly conserved. Orange frame shows residues constituting the ACT-I domain, blue the ACT-II, grey the PSP domain and yellow the CAP domain closing the active site. Green frames represent the highly conserved residues among PSP from various organisms (human, mammals, etc.). Structure of *M. avium* (3P96) with domain ACT-I in orange, ACT-II in blue, the linker between two ACT domains in red, PSP catalytic domain in grey, linker between PSP domain and ACT-II in green (**b**). The magnesium within catalytic core is in green and the highly conserved residues stabilizing it are highlighted. The α helix cap closing the active site is in yellow.

The overall sequence identity between phosphatases is often very low but members of this family can be identified by three highly conserved catalytic motifs containing catalytic residues [99]. Motif I is hhhDxDx(T/V)(L/V)h (h is a hydrophobic amino acid and x can be any amino acid) and contains the aspartate residue used for nucleophilic attack [100]. Crystallographic studies showed that those aspartates residues can complex a magnesium ion essential for phosphatase activity. Motif II (hhhhhh(S/T)) orients the substrate for the nucleophilic attack. In motif III (Kx${}_{18-30}$(G/S)(D/S)x${}_{3-4}$(D/E)hhhh), a lysine stabilizes the negative charge of the reaction intermediate. Aspartate/glutamate residues of this motif complex the magnesium ion. Those motifs can be found in MtSerB2, as shown in Figure 11a.

Structurally speaking, SerB2, like all HAD, possesses a Rossman-like α,β-core domain with 4 loops containing all 3 motifs. They constitute the phosphoserine phosphatase (PSP) domain which catalyzes the reaction. They also possess a cap domain that closes the active site in order to specifically recognize substrate. Depending on the differences between structure of cap domains, HAD can be divided in subfamilies. In subfamily I, cap is a small α-helical domain between Motif I and II. In the second subfamily, cap can be a mixture of α helices and β strands usually found between Motifs II and III. Cap can be quite dynamic and control the opened and closed conformations of the enzyme, knowing that the reaction usually occurs when the protein is in the closed conformation [93,101]. SerB2 possesses a small type I cap domain.

In contrast to other enzymes from the serine pathway (SerA and SerC), the crystal structure of SerB2 is still unknown. *Mycobacterium avium* phosphoserine phosphatase (MaSerB) is the closest crystallized counterpart. Indeed, SerB2 is highly conserved in other mycobacteria such as *Mycobacterium avium* (Ma) and *leprae* (Ml, Figure 11b). The sequence identity is 84% between MtSerB2 and MaSerB and 85% between MtSerB2 and MlSerB. SerB of *M. avium* was crystallized by Seattle Structural Genomics Centre for Infectious Disease (SSGCID) in 2010 (PDB: 3P96; Figure 11b) [102]. Since then, eight other structures have been deposited on the Protein Data Bank. MaSerB is then a good candidate to model interactions taking place between SerB2 and inhibitors or assist fragment-based design.

MtSerB2 has two ACT amino acid-binding domains in the N-terminal position. These domains are highly conserved allosteric domains used that regulate the enzyme activity in presence of a high concentration of reaction product. ACT domains have the so-called ferredoxin-like βαββαβ scaffold [103]. Kinetic studies performed by Grant [104] show that serine, the product of the reaction, acts as a partial competitive inhibitor, meaning it can interact with the active site (e.g., classical dead-end inhibitor), but also with ACT domains in a quite efficient manner (Ki = ~19 μM). Mutants D15/E33A are less sensitive to serine (Ki = ~6700 μM) showing that these residues are involved in this regulatory mechanism. Crystal structure deposited with serine bound at ACT domains (PDB: 5JLP) supports this result since D15 directly interacts with the amino group of serine. Interestingly, human phosphoserine phosphatase is only constituted of the PSP domain and targeting the ACT domains with small inhibitors may be a way to achieve selective inhibition of MtSerB2.

#### Inhibitors of Phosphatases

**Figure 12:**
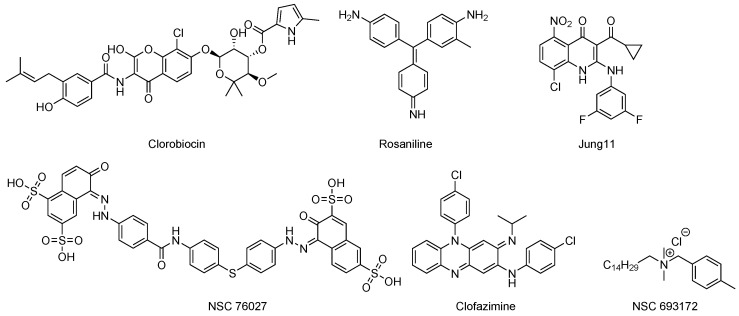
Current reported inhibitors of SerB2 or close counterparts.

The first inhibitors of SerB2 were described by Arora et al. [57] and were found by high throughput screening of 2300 compounds. Best primary hits were clorobiocin (an anti-bacterial agent) and rosaniline (Figure 12). They could inhibit SerB2 as well as mycobacterial growth with no toxicity against THP-1 cells. Another inhibitor, NSC 76027, is very potent in vitro but display poor activity (MIC${}_{99}$ = 150 μM) against *Mycobacterium tuberculosis* H${}_{37}$Rv strain. NSC 693172 is also a good inhibitor of Mtb growth (MIC${}_{99}$ = 12.5 μM) but highly toxic against THP-1 cells. They were tested against human PSP, and only rosalinine inhibits both proteins.

Clofazimine (Figure 12) is a riminophenazine anti-leprosy drug used as a “last resort” against MDR and XDR TB. The inhibition of SerB2 by clofazimine is competitive with a Ki of 2.74 ± 0.016 μM [9]. Clofazimine diminishes the ability of SerB2 to induce cytoskeletal rearrangement and to dephosphorylate other proteins.

3-Acyl-2-phenylamino-1,4-dihydroquinolin-4-one derivatives can inhibit SerB653, a close counterpart of SerB2 (36.2% identity and 54.8% similarity). The best compound Jung11 (Figure 12) has a Ki of 1.0 μM and a MIC of 14 nM against *Porphyromonas gingivalis* [105,106]. Such inhibitors could be effective against other bacteria such as *Streptococcus pneumoniae*. Surprisingly, they inhibit bacterial growth even better than they inhibit the enzyme of interest, suggesting that either molecules quickly accumulate within the bacteria leading to rapid inhibition of PSPs or they have more than one mechanism of action [107].

Informations about the state of drug development of those different inhibitors could not be found. Clofazimine efficiency is evaluated on MDR-strains [24].

## 4. Summary

In summary, MDR and XDR TB are on the rise despite many efforts to diminish their prevalence [20]. Many clinical trials are ongoing but few new therapeutic compounds are in the pipeline [24]. Three compounds are in Phase 3 of clinical trials: Bedaquiline, Delamanid, and Pretomanid. Resistance has already been observed for Bedaquiline and Delamanid [30,31]. There is thus an urgent need for new compounds. Target-based design is claimed to be the most efficient way to achieve this goal by experts [33].

Decoding of Mtb genome by Cole et al. [36] and experiments from Sassetti et al. [37] showed that the serine pathway is required for bacterial growth [85]. SerA1 catalyzes the first step of the serine pathway in a reversible manner [63]. It is specific for D-configurated substrates and was crystallized for the first time in 2005 [65]. The enzyme is made of four distinct domains with ACT and ASB domains being regulatory [67]. The latter can rotate by 180° with respect to nucleotide and substrate binding domains. It was suggested that this rotation is due to cofactor binding and release. Kinetic experiments shows that L-serine and phosphohydroxypyruvate act as allosteric inhibitors of SerA1 when they bind ACT and ASB. Residues interacting with those ligands are mutated in human PHGDH. Therefore, small polar and charged molecules interacting with those allosteric domains could be designed in order to inhibit the bacterial enzyme. No inhibitors specific for MtSerA1 are described. Inhibitors of human PHGDH could be a good starting point for fragment-based lead generation of compounds active against Mtb [69].

SerC catalyzes the second reversible step and is not very well described in the literature. Its structure was determined in 2012 by Coulibaly et al. [56] at a resolution of 1.5 Å. No inhibitors of SerC have been reported so far, even if this enzyme is considered as a potential drug target candidate [48].

SerB2 catalyzes the third non-reversible step of the serine pathway. Shree et al. [9] showed that Mtb phosphoserine phosphatase (SerB2) is secreted within macrophages causing cytoskeletal rearrangements and immune suppression. Protein–protein interactions take place between MtSerB2, cofilin, p38, and NFκB. SerB2 belongs to the HAD superfamily and is highly conserved among different mycobacteria [102]. Its crystallographic structure is not determined yet, but its closest homolog (MaSerB, 84% identity) was crystallized in 2010. It could be used in order to get structural knowledge on MtSerB2. This structure possess three large domains: the PSP domain which catalyzes the reaction and two small ACT regulatory domains. Grant [104] and Yadav [58] showed that L-serine inhibits the activity of SerB2 by interaction with ACT I domain. The ACT domains are not present in human phosphoserine phosphatase, which potentially opens the possibility to achieve selective inhibition [57].

Few inhibitors of SerB2 have been described in the literature. All have been found to be competitive inhibitors [9,57,105]. They are mostly active against bacteria, which can lead to the inhibition of macrophage cytoskelatal rearrangement and restore immune response [9]. Targeting the Mtb serine pathway is thus a promising approach when designing new antitubercular compounds. 

## Abbreviations

The following abbreviations are used in this manuscript: MDR: Multi-Drug Resistant; XDR: Extensively Drug-Resistant; Mtb: *Mycobacterium tuberculosis*; TB: Tuberculosis; HIV: Human Immunodeficiency Virus; WHO: World Health Organization; RR: Rifampin-Resistant; E.R., European Region; W.P.R., Western Pacific Region; E.M.R., Eastern Mediterranean Region; A.R., Americas Region; S.E.A.R, Southeast Asia Region; Af.R., Africa Region; DS-TB: Drug Susceptible Tuberculosis; GSK: GlaxoSmithKline; 3-PGA, 3-phosphoglycerate; 3PHP, 3-phosphohydroxypyruvate; 3-PS, 3-Phospho-l-Serine; PGDH, Phosphoglycerate dehydrogenase; PSAT, Phosphoserine aminotransferase; PSP, Phosphoserine phoshatase; SHMT, Serine hydroxymethyltransferase; Pi, ${\mathrm{PO}}_{4}^{3-}$; TCA, tricarboxylic acid cycle; SBD: Substrate Binding Domain; NBD: Nucleotide Binding Domain; ASB: Allosteric Substrate Binding Doamin; ACT: Aspartate kinase, Chorismate mutase and TyrA domain; NAD^+^: Nicotinamide Adenine Dinucleotide; PLP: Pyridoxal 5′-phosphate; IL-8: Interleukin 8; HAD: Haloacid Dehalogenase; MIC: Minimum Inhibitory Concentration; Ki: Inhibition constant.
